# zBMI scales with multispectral alterations in the neural oscillatory dynamics serving verbal working memory in youth

**DOI:** 10.1162/IMAG.a.78

**Published:** 2025-07-09

**Authors:** Thomas W. Ward, Abraham D. Killanin, Danielle L. Rice, Grace C. Ende, Erica L. Steiner, Anna T. Coutant, Christine M. Embury, Vince D. Calhoun, Yu-Ping Wang, Julia M. Stephen, Elizabeth Heinrichs-Graham, Tony W. Wilson

**Affiliations:** Institute for Human Neuroscience, Boys Town National Research Hospital, Boys Town, NE, United States; Center for Pediatric Brain Health, Boys Town National Research Hospital, Boys Town, NE, United States; Department of Pharmacology & Neuroscience, Creighton University, Omaha, NE, United States; College of Medicine, University of Nebraska Medical Center, Omaha, NE, United States; Tri-Institutional Center for Translational Research in Neuroimaging and Data Science (TReNDS), Georgia State University, Georgia Institute of Technology, and Emory University, Atlanta, GA, United States; Department of Biomedical Engineering, Tulane University, New Orleans, LA, United States; Mind Research Network, Albuquerque, NM, United States

**Keywords:** magnetoencephalography, development, obesity, theta, alpha

## Abstract

Pediatric obesity is one of the most serious public health issues the world faces today. Deleterious behavioral effects scaling with obesity and body mass have been demonstrated in cognitive tasks in children and adults, yet the neural oscillatory dynamics underlying these effects remain largely unstudied. In this study, 88 youth (6–13 years old) performed a verbal working memory task during high-density magnetoencephalography (MEG). The MEG data were transformed into the time–frequency domain and oscillatory responses during encoding and maintenance phases were imaged separately using a beamformer approach. Each participant’s height, weight, sex, and age were used to create age- and-sex-adjusted body mass index (i.e., zBMI) measures and BMI-for-age percentiles. Whole-brain correlation maps were examined for effects of body mass on neural dynamics serving encoding and maintenance. According to the BMI-for-age percentiles, 21 subjects were classified as overweight/obese. Behaviorally, our results indicated that task accuracy and reaction time were strongly correlated with age but not zBMI, such that older youth were faster and more accurate than their younger peers. Neural oscillatory activity in the theta (4–7 Hz) and alpha (8–12 Hz) range scaled with zBMI in several left-hemispheric regions across occipital, temporal, and frontal cortices. During the encoding phase, elevated zBMI was associated with *weaker* theta and *stronger* alpha oscillations. In the maintenance phase, higher zBMIs were associated with *stronger* alpha oscillatory activity. Taken together, these results suggest that oscillatory dynamics in brain regions central to working memory processing are vulnerable to deviations from normal body mass in specific spectral bands. The stronger alpha and weaker theta oscillations may point to opposing compensatory and deleterious effects of elevated zBMI on working memory, respectively.

## 1 Introduction

Pediatric obesity is a global issue that the World Health Organization has deemed one of the most significant public health problems of the 21^st^ century (World Health Organization, 2020). In fact, rates in the United States have nearly quadrupled over the past five decades (Fryar et al., 2020). There are a range of environmental, societal, and policy factors that have contributed to these persistent increases in obesity rates. Some of the most significant include the combination of increased access and decreased cost of calorie-rich, nutrient-poor foods, coupled with the reduced access and increased cost of healthy foods (Bevel et al., 2023; Bodor et al., 2010). Obesity is a chronic disease and a risk factor for the development of metabolic and cardiovascular disorders (Mandviwala et al., 2016; Pi-Sunyer, 2009; Poirier et al., 2006; Rippe et al., 1998). More broadly, obesity is associated with a higher incidence of comorbid diseases and reduced life expectancy, particularly when onset occurs earlier in life (Fontaine et al., 2003; Tsur et al., 2023). There is also a growing literature connecting obesity to cognitive and brain dysfunction (Boeka & Lokken, 2008; Laurent et al., 2020; Sargénius et al., 2017; Wu et al., 2017).

Working memory is the process of loading and maintaining information in temporary memory stores (Baddeley, 2010) and is known to be strongly predictive of future academic and career success (Best et al., 2011; Visu-Petra et al., 2011). The predominant cognitive theory on working memory is Baddeley and Hitch’s multicomponent model, which divides working memory into three separate processes: the phonological loop, visuospatial sketchpad, and central executive. In this framework, the phonological loop and visuospatial sketchpad are involved in the short-term retention of verbal and spatial components, respectively, while the central executive acts primarily as an attentional control system, dividing and switching attention as needed (Baddeley, 2002; Baddeley & Hitch, 1974; D’Esposito et al., 1995). Such executive functions are known to undergo a protracted period of development (Luna et al., 2004, 2015) that can be modulated by external factors, including socioeconomic status, trauma, inflammation, and obesity (DePrince et al., 2009; Hackman et al., 2015; Lee et al., 2016; Ronan et al., 2020). For example, obesity has been consistently linked to behavioral deficits in working memory performance among both adults and youth (Christina et al., 2021; Coppin et al., 2014; Laurent et al., 2020; Wu et al., 2017). Evidence that chronic inflammation impacts oscillatory activity supporting cognitive processes (Dietz et al., 2023; Spooner et al., 2021) lends support to hypotheses that the effects of elevated body mass on neural activity may be due to, or at least influenced by, the chronic low-grade inflammatory state associated with obesity (Kullmann et al., 2020; Naznin et al., 2015). Nonetheless, the neural mechanisms underlying these deficits have been minimally studied and remain unclear, in both adult and pediatric samples.

Working memory is typically thought to occur in three separate phases: (1) encoding, where the relevant stimuli are loaded into a temporary storage buffer, (2) maintenance, where stimuli are maintained in memory through rehearsal or other strategies, and (3) retrieval, where the information is recalled to be used toward some goal. The neural oscillatory activity underlying these separate phases has been widely characterized in both children and adults (Diedrich et al., 2025; Embury et al., 2019; Heinrichs-Graham & Wilson, 2015; Killanin et al., 2022, 2024a, 2024b, 2025; McKeon et al., 2023; Proskovec et al., 2016, 2018; Wiesman et al., 2016). Broadly, alpha band activity (i.e., oscillatory activity falling between 8 and 16 Hz) has been shown to be critical for accurate and efficient encoding and maintenance in several investigations in adults. Decreases in alpha power relative to baseline are thought to reflect the active processing of information in the underlying neural populations (Crespo-Garcia et al., 2013; Klimesch, 2012). Beginning in the occipital cortices in the encoding phase, this response gradually progresses to anterior regions throughout maintenance in left parietal, temporal, and prefrontal regions (Embury et al., 2023; Heinrichs-Graham & Wilson, 2015; Killanin et al., 2024a, 2024b; Koshy, Wiesman, Proskovec, et al., 2020; Springer et al., 2023; Wiesman, Christopher-Hayes, et al., 2021). As these alpha decreases shift more anterior toward temporal and frontal cortices during maintenance, strong increases in alpha power emerge in the occipital lobe, which is thought to reflect the active inhibition of incoming, task irrelevant visual information (Bonnefond & Jensen, 2012; Embury et al., 2018, 2023; Gutteling et al., 2022; Heinrichs-Graham & Wilson, 2015; Koshy, Wiesman, Spooner, et al., 2020; Proskovec et al., 2016). These responses have also been observed in children and adolescents, though with greater variability largely attributed to inefficient recruitment of maturing brain networks serving working memory processes (Embury et al., 2019; Finn et al., 2010; Jolles et al., 2011; Killanin et al., 2024a).

However, as previously mentioned, the relationship between obesity and oscillatory activity underlying working memory is unclear. Very few studies have specifically investigated this relationship in adults (Hege et al., 2013; Stingl et al., 2012), and to our knowledge, none to date has examined how deviations from normal body mass are associated with the neural dynamics serving working memory in youth. Further, only one study has examined the relationship between body mass and oscillatory activity in youth performing any cognitive task (i.e., abstract reasoning; Ward et al., 2024). Thus, in this study, we examined how body mass may impact the neural oscillatory dynamics serving working memory processing. Body mass index (BMI) percentiles standardized for age and sex using the United States Centers for Disease Control and Prevention (CDC) growth charts (e.g., zBMI) are typically used to quantify obesity in youth, as the relationship between total body mass and adiposity changes dramatically during development (Chung, 2015). In sum, we utilized high-density dynamic functional mapping with MEG, age- and-sex-adjusted body mass indices, and whole-brain statistical analyses to investigate the relationship between body mass and the neural dynamics serving verbal working memory processes in a sample of typically developing youth. We hypothesized that zBMI would be associated with altered alpha and theta activity during encoding and maintenance periods during a verbal working memory paradigm.

## 2 Methods

### 2.1 Participants

The sample included a total of 106 participants between the ages of 6 and 13 years old who were recruited during year 1 of a 5-year accelerated longitudinal study and had completed the verbal working memory paradigm during MEG, structural MRI, and height and weight measurement. It should be noted that due to the accelerated longitudinal design of the Developmental Multimodal Imaging of Neurocognitive Dynamics (DevMIND) study (R01-MH121101), 9- and 10-year olds were not enrolled. Thus, our sample consisted of 6-to 8-year olds and 11- to 13-year olds. Potential participants were recruited from the Omaha, Nebraska metropolitan area. Exclusion criteria for the study included any diagnosed neurological or psychiatric disorder, any medical illness associated with CNS dysfunction, history of head trauma, current substance use, and standard MEG/MRI contraindications (e.g., metallic implants that could impede MEG or MRI data acquisition). All study procedures were approved by the local institutional review board (IRB) and all ethical regulations relevant to human research participants were followed in accordance with the Declaration of Helsinki. Before participating, the child and the parent or legal guardian provided written informed assent and consent.

### 2.2 Standardized body mass index (zBMI)

Each participant’s height and weight were measured at the study visit and were used to compute a body mass index (BMI) score by dividing their weight in kilograms by the square of their height in meters. Standardized BMI scores were created based on sex and age (in months) using the CDC growth charts, which contain 10 smoothed percentiles (between the third and 97^th^) of BMI for children and adolescents between the ages of 24 and 240 months (Flegal et al., 2009; Kuczmarski et al., 2002). These estimates from the growth charts were used to derive the parameters of lambda (L, the power transformation to achieve normality), mu (M, mean), and sigma (S, coefficient of variation) for each participant; these parameters can then be used to convert measurements into z-scores. The LMS parameters for BMI were derived from equations for smoothed percentiles (Flegal et al., 2009; Flegal & Cole, 2013) based on Cole’s LMS method (Cole, 1990; Cole & Green, 1992). Estimates of these three parameters allow the BMI of youth to be expressed as a z-score (zBMI) relative to children of the same sex and age in the CDC growth charts using the following **Equation 1** (Cole et al., 2000), in which higher zBMI values are indicative of higher age- and sex-adjusted BMIs:

$$zBMI=\left[{\left(\frac{BMI}{M}\right)}^{L}-1\right]÷\left(L\times S\right).$$ (**1**)

Using the cumulative response function of the standard normal distribution (*ϕ*), zBMI values can be used to obtain a CDC BMI-for-age percentile for each participant through **Equation 2**:

$$BMI_{percentile}=\;\phi \left(zBMI\right)\;\times \;100.$$ (**2**)

### 2.3 Experimental paradigm

**Fig. 1.**
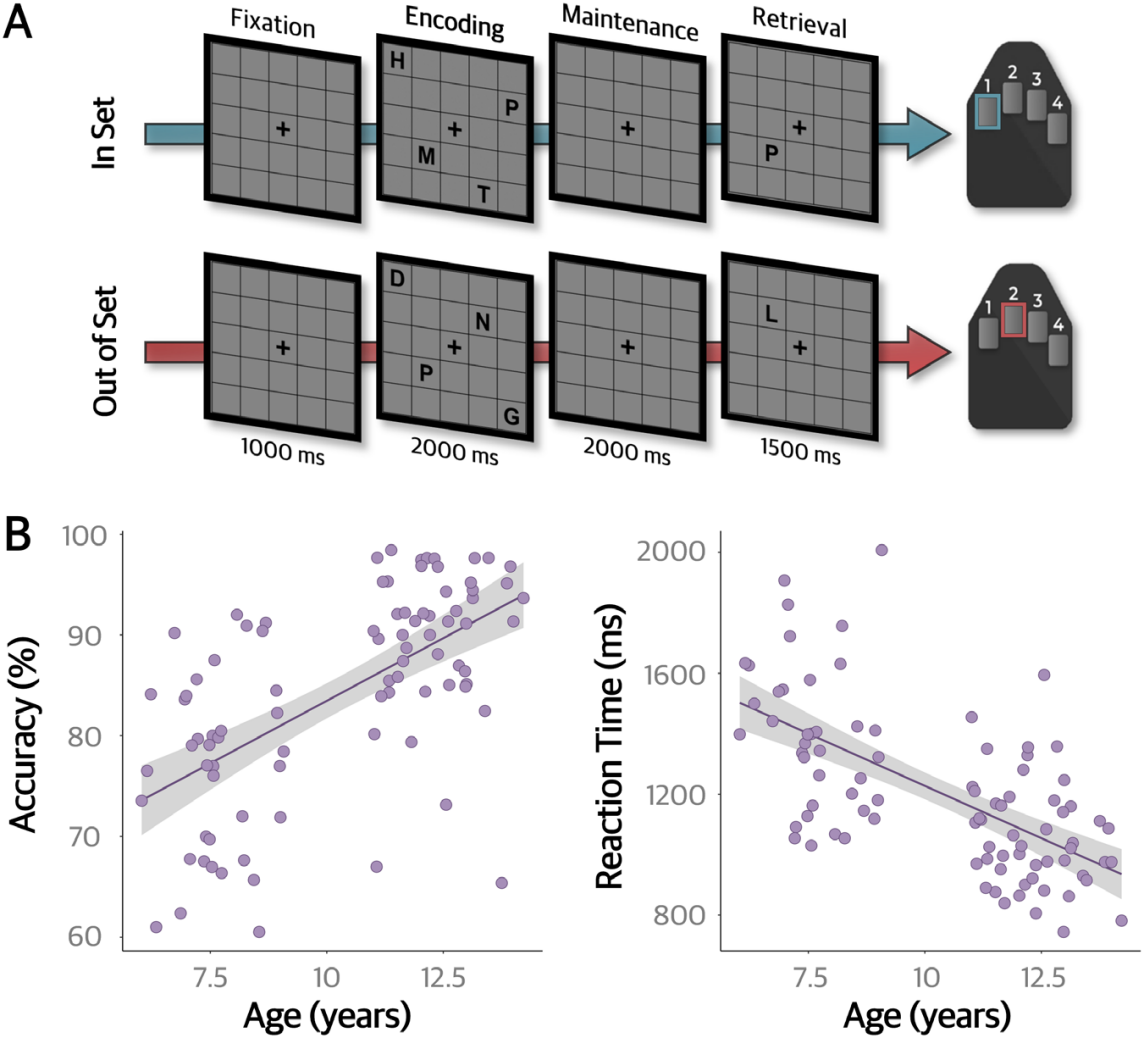
Task paradigm and behavioral results. (A) Example in-set (top) and out-of-set (bottom) trial of the modified Sternberg working memory task with associated correct response. Participants were instructed to view a fixation cross in an empty 5 x 5 grid for 1000 ms. Next, four consonants appeared for 2000 ms and participants had to encode the letters into memory (independent of spatial location). These letters then disappeared, and participants had to remember the letters for a 2000 ms maintenance period. Finally, a single probe letter appeared and if it was present in the encoding set (i.e., in-set), participants responded with their right index finger. If the probe was not present in the encoding grid (i.e., out-of-set), participants responded with their right middle finger. (B) Behavioral results for the working memory task. Accuracy and reaction time were significantly correlated with chronological age (accuracy: *r* = .60, *p* < .001; reaction time: *r* = -.64, *p* < .001), such that older youth performed better on the task.

We used a modified Sternberg working memory task (Sternberg, 1966) during MEG recording that was based on the design used in previous studies of adults and youth in our laboratory (Arif et al., 2024; Killanin et al., 2024a, 2024b, 2025; Proskovec et al., 2019; Wilson et al., 2017). Participants were instructed to rest their right hand on a five-finger button pad and view a centrally presented fixation cross embedded in an otherwise empty 5 x 5 grid for 1000 ms. Following this fixation period, four consonants appeared at fixed locations within the grid for 2000 ms (i.e., encoding period). The letters then disappeared, with only the empty grid and fixation cross remaining for another 2000 ms during which participants maintained the encoded consonants in memory. Following this maintenance phase, a probe letter appeared on the grid for 1500 ms (i.e., retrieval period) and participants responded via a button press with their right index finger if the probe was present in the encoding array (i.e., in-set), or middle finger if the probe was absent from the encoding array (i.e., out-of-set). Participants were instructed to only attend to the letters themselves, and to ignore their positions within the grid. The experiment consisted of 128 pseudo-randomized trials split equally between in- and out-of-set trials, resulting in approximately 15 min of recording time (Fig. 1A).

### 2.4 MEG and MRI data acquisition

Functional MEG data were collected using an Elekta/MEGIN MEG system (Helsinki, Finland) equipped with 306 sensors (204 planar gradiometers, 102 magnetometers) using a 1 kHz sampling rate and an acquisition bandwidth of 0.1–330 Hz in a 1-layer magnetically shielded room with active shielding engaged. Before MEG acquisition, four coils were attached to the participant’s head and localized along with fiducial and scalp surface points using a three-dimensional digitizer (Fastrak, Polhemus Navigator Sciences, Colchester, Vermont). Once the participants were positioned for MEG recording, an electric current with a unique frequency label (e.g., 322 Hz) was fed to each of the four coils, thus inducing a measurable magnetic field, thereby allowing each coil to be localized in reference to the MEG sensor array throughout the recording session.

Structural T1-weighted images were collected using a Siemens Prisma 3T scanner with a 32-channel head coil. Each participant’s MRI data underwent AC/PC alignment, inhomogeneity correction, segmentation, surface reconstruction, and transformation into standardized Talairach space. Each participant’s MEG data were co-registered with their MRI data using BESA MRI (v3.0). After source imaging, each subject’s functional images were also transformed into standardized space using the transform previously applied to the structural MRI volume, and spatially resampled.

### 2.5 Time–frequency transformation and statistics

MEG data were subjected to environmental noise reduction and corrected for head motion using the signal space separation method with a temporal extension (Taulu & Simola, 2006). Eye blinks and cardiac artifacts were removed from the data using signal space projection (SSP), which was accounted for during source reconstruction (Uusitalo & Ilmoniemi, 1997). The time series was then divided into 6500 ms epochs, including a baseline window from -400 to 0 ms, with the onset of the encoding grid defined as 0 ms. Only the correct trials were used for analysis. Epochs containing artifacts were then rejected based on a fixed threshold method that was set per participant and supplemented with visual inspection. Briefly, in MEG, the raw signal amplitude is strongly related to the distance between the brain and the MEG sensor array, as the magnetic field strength falls off sharply as the distance from the current source (i.e., brain) increases (i.e., approximately 1/distance^2^). Thus, to account for this and other sources of variance across participants, we used the individualized threshold based on the signal distribution for both amplitude (*M* = 2141.2 fT/cm, *SD* = 918.55) and gradient (*M* = 536.7 fT/(cm × ms), *SD* = 262.12) to reject artifacts. Note that an average of 87.19 (*SD* = 11.92) correct trials per participant remained for further analysis and the number of accepted trials was not significantly correlated with age (*r* = .18, *p* = .09) or zBMI (*r* = .009, *p* = .93).

Artifact-free epochs were transformed into the time–frequency domain using complex demodulation (Kovach & Gander, 2016; Papp & Ktonas, 1977). The resulting spectral power estimations per sensor were averaged across trials, generating time–frequency plots of mean spectral density. The sensor-level data were then normalized per time–frequency bin using the respective bin’s baseline power, which was calculated by averaging the power during the 400 ms baseline period. The specific time–frequency bins used for source reconstruction were determined using a mass univariate approach based on the general linear model. To reduce the risk of false-positive results while maintaining reasonable sensitivity, a two-stage procedure was followed to control for Type-1 error. In the first stage, two-tailed paired-sample *t*-tests against baseline were conducted on each data point, and the output spectrogram of *t*-values was thresholded at *p* < .05 to define time–frequency bins containing potentially significant oscillatory deviations across all participants. In stage 2, time–frequency bins that survived the threshold were clustered with temporally and/or spectrally neighboring bins that were also above the threshold (*p* < .05), and a cluster value was derived by summing the *t*-values of all data points in the cluster. Nonparametric permutation testing was then used to derive a distribution of cluster values, and the significance level of the observed clusters (from stage 1) was tested directly using this distribution (Ernst, 2004; Maris & Oostenveld, 2007). For each comparison, 10,000 permutations were computed. Based on these analyses, the time–frequency windows containing significant oscillatory events relative to baseline across all participants were subjected to beamforming. For further details on our data processing pipeline, see Wiesman and Wilson (2020).

### 2.6 MEG source imaging and statistical analyses

Oscillatory neural responses were imaged using the dynamic imaging of coherent sources (DICS) beamformer (Gross et al., 2001; Van Veen et al., 1997), which utilizes spatial filters in the time–frequency domain to calculate voxel-wise source power for the entire brain volume. The single images were derived from the cross-spectral densities of all combinations of MEG gradiometers averaged over the time–frequency range of interest and the solution of the forward problem for each location on a grid specified by input voxel space. Following convention, we computed noise-normalized source power for each voxel per participant using active (i.e., task) and passive (i.e., baseline) periods of equal duration and bandwidth (Hillebrand et al., 2005) at a resolution of 4.0 x 4.0 x 4.0 mm. Such images are referred to as pseudo-*t* maps, with units (pseudo-*t*) reflecting noise-normalized power differences (i.e., active versus passive) per voxel. To assess the neuroanatomical basis of the significant oscillatory responses identified through the sensor-level analysis, grand-average whole-brain pseudo-*t* maps were computed, excluding outliers. MEG preprocessing, coregistration, and imaging used the Brain Electrical Source Analysis (BESA Research v7.1, Statistics v2.1, MRI v3.0) software.

We assessed the relationship between zBMI and oscillatory responses using voxel-wise correlations with the participant-level source images (Ward et al., 2024) for each time–frequency window identified in the sensor-level analysis. Whole-brain correlations were performed using custom in-house MATLAB scripts (v2018b; MathWorks, Natick, MA). A stringent *p*-value threshold of *p* < .005 was used in conjunction with a cluster threshold of 9 contiguous voxels (i.e., >550 mm^3^ of neural tissue) for the resultant statistical maps to account for multiple comparisons based on the theory of random Gaussian fields (Poline et al., 1995; Worsley et al., 1996, 1999).

Correlations between task performance (i.e., accuracy, reaction time), age, and zBMI were performed in IBM SPSS Statistics (SPSS Statistics for Windows, Version 25.0.; Armonk, NY: IBM Corp.). Data plots were created in R using ggplot2 (Wickham, 2016). Finally, to reduce the impact of outliers on statistical analyses, participants with values that were 2.5 SDs above or below the respective sample mean were excluded.

### 2.7 Exploratory brain–behavior correlation analysis

We conducted an exploratory analysis investigating the functional relevance of the clusters identified in the whole-brain analyses. Specifically, we performed partial correlations between performance metrics on the verbal working memory task (i.e., accuracy and reaction time) and oscillatory power at the peak voxel of each cluster, controlling for zBMI. This analysis was conducted in IBM SPSS Statistics.

## 3 Results

### 3.1 Participant characteristics and behavioral results

Of the 106 participants, 16 were excluded from analysis due to poor task performance (i.e., accuracy < 60%) and 2 were excluded due to incomplete height and weight data. The remaining sample of 88 (39 female) youth successfully completed the verbal working memory task and had complete data on height, weight, age, and sex. The sample had a mean age of 10.30 years (SD = 2.47 years) and a mean zBMI of 0.36 (*SD* = 0.96). Females had a mean zBMI of 0.50 (*SD* = 1.04), and males had a mean zBMI of 0.20 (*SD* = 0.90). The distribution of zBMI values across the sample was approximately normal, with a skewness of 0.19 and kurtosis of -0.05 (Supplementary Fig. 1). Based on the CDC’s BMI-for-age percentiles (Flegal et al., 2009; Kuczmarski et al., 2002), 2 subjects were classified as underweight (<5^th^ percentile), 65 as healthy weight (between the 5^th^ and 85^th^), 13 as overweight (between the 85^th^ and 95^th^), and 8 with obesity (≥95^th^). There were no differences in age between males and females (*t* = 0.64, *p* = .52).

Overall, participants performed reasonably well on the task, with a mean accuracy of 84.2% (*SD* = 10.2%) and reaction time of 1206.86 ms (*SD* = 268.0 ms). Neither accuracy (*t* = -0.46, *p* = .65) nor reaction time (*t* = -1.19, *p* = .24) differed by sex. There were significant correlations with age in both measures, such that older participants were more accurate (*r* = .60, *p* < .001) and had faster reaction times (*r* = -.64, *p* < .001) than their younger peers (Fig. 1B). zBMI was not associated with behavioral performance (accuracy: *r* = -.04, *p* = .689; reaction time: *r* = -.05, *p* = .66).

### 3.2 Sensor- and source-level analysis

**Fig. 2.**
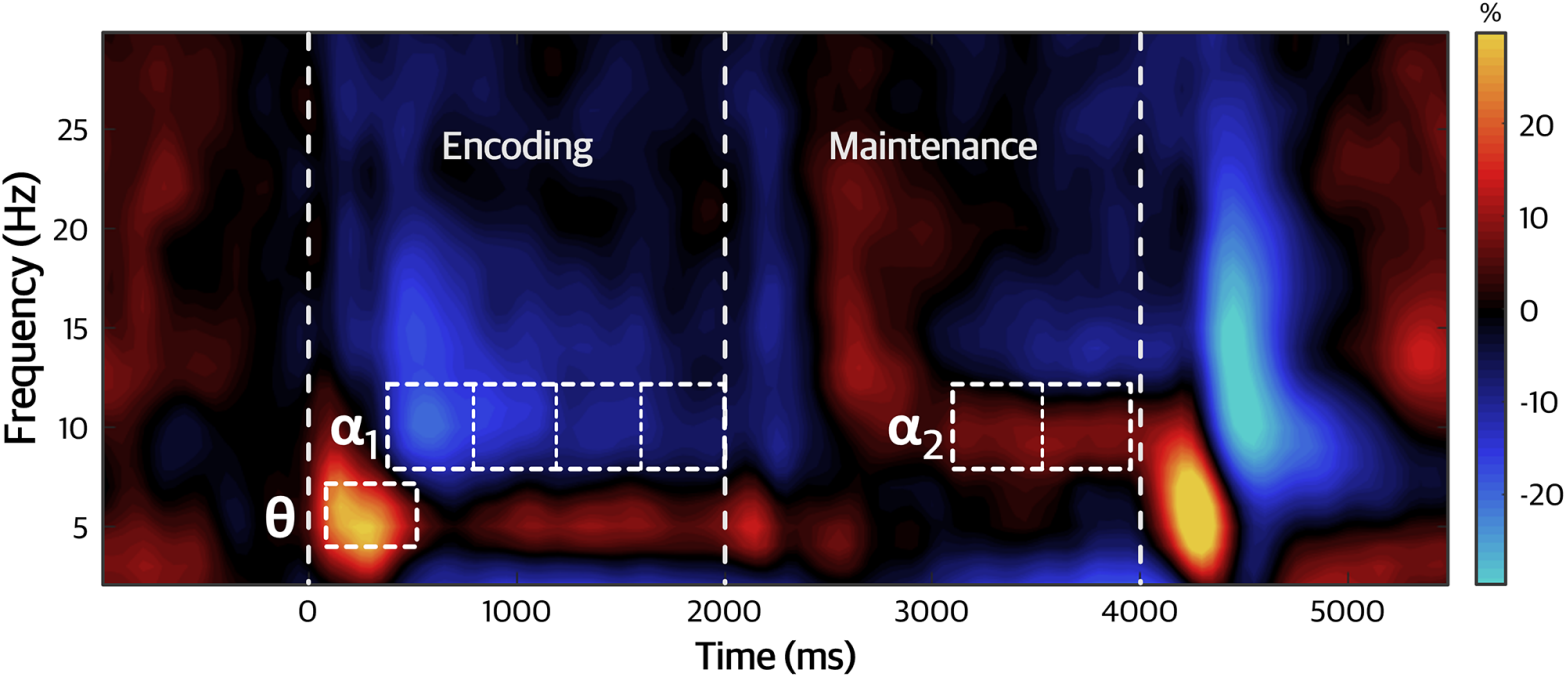
Time–frequency spectrogram. Grand-averaged time–frequency spectrogram across all participants from a representative sensor near the parietal cortex (i.e., MEG2043). Time (ms) is displayed on the x-axis (0 ms: encoding onset, 2000 ms: maintenance onset) with frequency (Hz) on the y-axis. Signal power, shown in the color scale, is expressed as percent change from baseline. Time–frequency windows and sub-windows for beamforming are outlined with white dashed boxes.

Sensor-level time–frequency analyses across all participants revealed significant alpha and theta responses in parieto-occipital and other sensors in both encoding and maintenance phases (Fig. 2; *p* < .0005, corrected). The alpha encoding response had a bandwidth of 8–12 Hz and was divided into 4 consecutive 400 ms windows spanning from 400 to 2000 ms. The theta encoding response encompassed 4–7 Hz, from 100 to 500 ms. Finally, the alpha maintenance response, also from 8 to 12 Hz, was divided into two 400 ms windows between 3100 and 3900 ms.

To identify the anatomical regions generating the alpha and theta responses, we imaged the significant time–frequency windows from the sensor-level analysis. This revealed robust decreases in alpha (8–12 Hz) power relative to baseline from 400 to 2000 ms in bilateral occipital cortices that became weaker and more left-lateralized throughout the encoding period, along with increases in baseline-relative theta (4–7 Hz) power from 100 to 500 ms in parietal and occipital cortices. In the maintenance period, we observed significant increases in alpha (8–12 Hz) power in the parietal cortex from 3100 to 3900 ms.

### 3.3 Whole-brain effects of zBMI on working memory encoding and maintenance

**Fig. 3.**
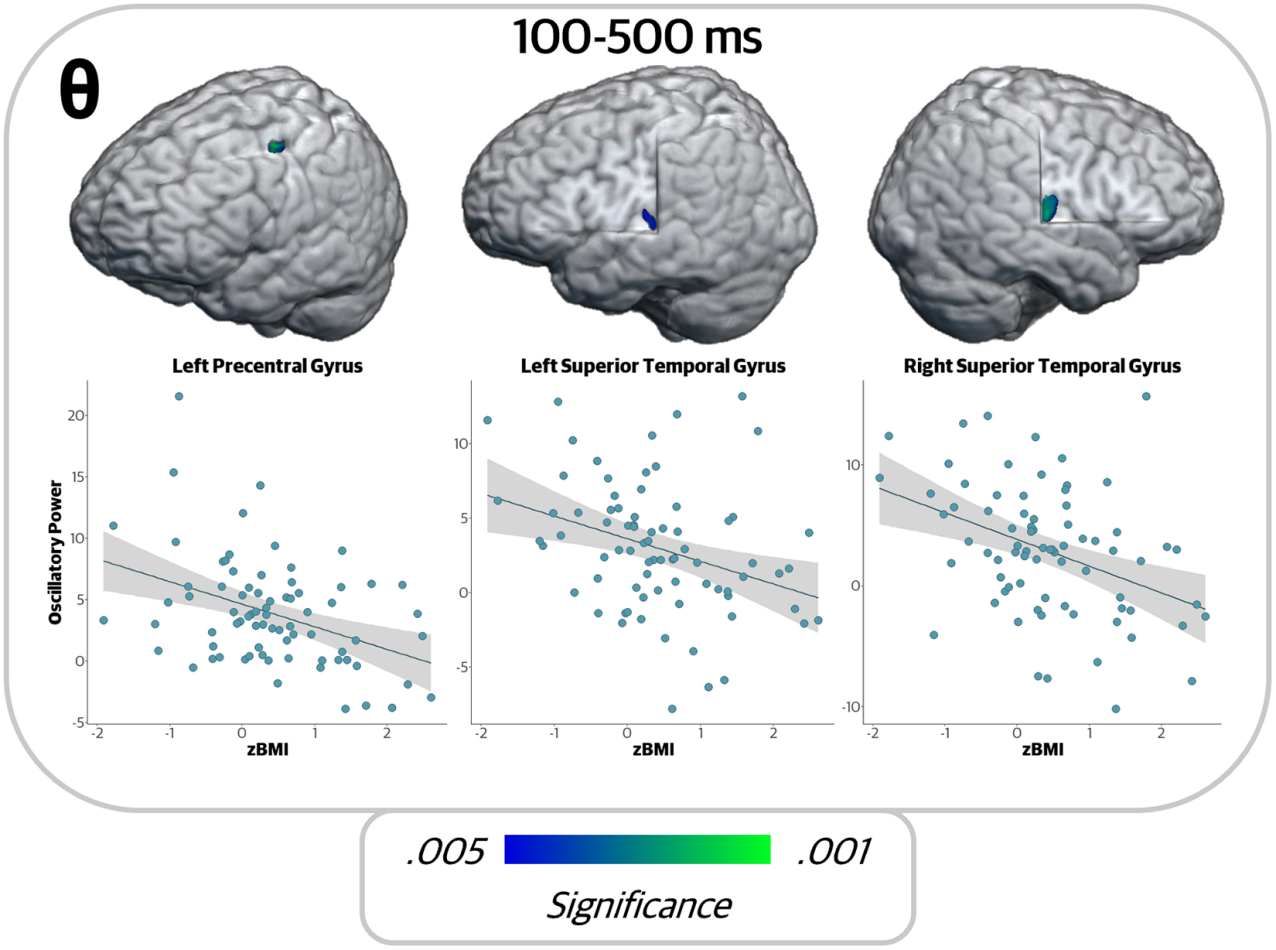
Higher zBMI was associated with weaker theta oscillatory power during encoding. Whole-brain correlation maps and corresponding scatterplots showing the relationship at the peak voxel between theta oscillations during encoding and zBMI. The x-axis displays zBMI values and the y-axis represents oscillatory power in units of pseudo-*t*. In all regions, there was a negative relationship between zBMI and oscillatory theta power, such that theta decreased as zBMI increased.

Next, to identify the relationship between age- and sex-adjusted BMI and the neural dynamics serving working memory function, we conducted whole-brain correlations between the participant-level functional maps and zBMI in each time–frequency window of interest. In the theta encoding window (100 to 500 ms), zBMI was negatively correlated with theta oscillations in the left precentral gyrus (*r* = -.404, *p* < .001) and bilateral superior temporal gyri (left: *r* = -.398, *p* < .001; right: *r* = -.388, *p* = .003). Note that the *r-* and *p*-values reported throughout this manuscript are from the peak voxel of each cluster. In all three regions, increases in zBMI values were associated with reduced (i.e., a smaller increase in power relative to baseline) theta oscillations (Fig. 3).

**Fig. 4.**
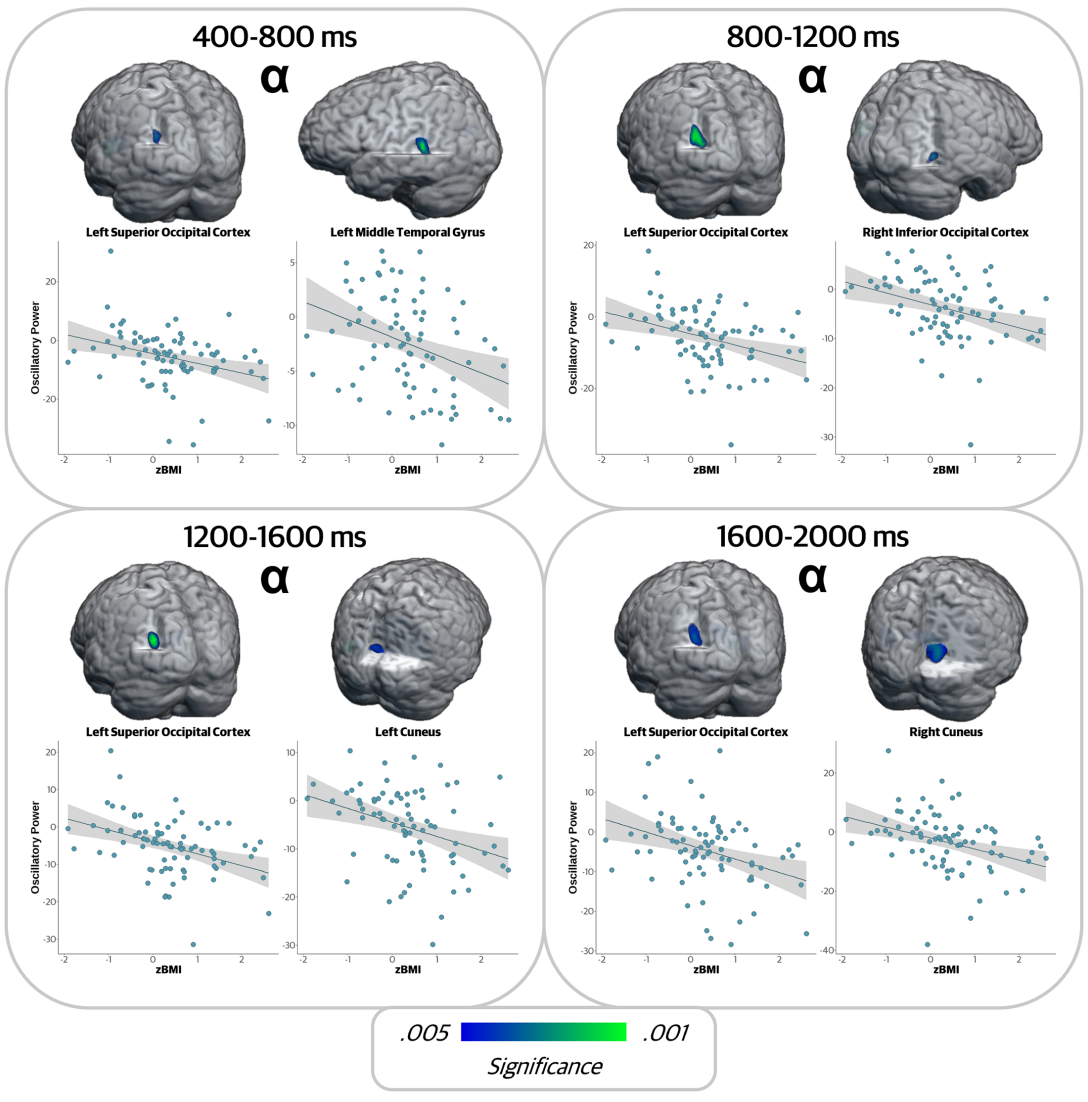
Higher zBMI was associated with stronger alpha oscillatory activity during encoding. Whole-brain correlation maps per time window and corresponding scatterplots showing relationships at the peak voxel per cluster between alpha oscillations during encoding and zBMI. The x-axis displays zBMI values and the y-axis represents oscillatory power in units of pseudo-*t*. In all regions, there was a negative relationship between zBMI and oscillatory power, such that as zBMI increased, alpha oscillations became stronger (i.e., more negative relative to baseline).

In the earliest alpha encoding window (400 to 800 ms), neural oscillations in the left superior occipital cortex and left middle temporal gyrus were negatively correlated with zBMI (*r* = -.335, *p* = .002; *r* = -.356, *p* = .001, respectively), indicating that alpha oscillations were stronger (i.e., greater decreases in power relative to baseline) in those with higher zBMI values (Fig. 4). Likewise, from 800 to 1200 ms, alpha oscillations in the left superior occipital cortex and right inferior occipital areas were also negatively correlated with zBMI (*r* = -.363, *p* < .001; *r* = -.352, *p* = .001, respectively). From 1200 to 1600 ms, alpha activity in the left superior occipital cortex and left cuneus was negatively correlated with zBMI (*r* = -.398, *p* < .001; *r* = -.343, *p* = .002, respectively). Finally, in the last alpha encoding window (1600 to 2000 ms), responses in the left superior occipital cortex and right cuneus were negatively correlated with zBMI (*r* = -.356, *p* = .001; *r* = -.365, *p* < .001, respectively). In all cases, the relationship at each region was such that elevated zBMIs were associated with stronger alpha oscillations (i.e., more negative relative to baseline).

**Fig. 5.**
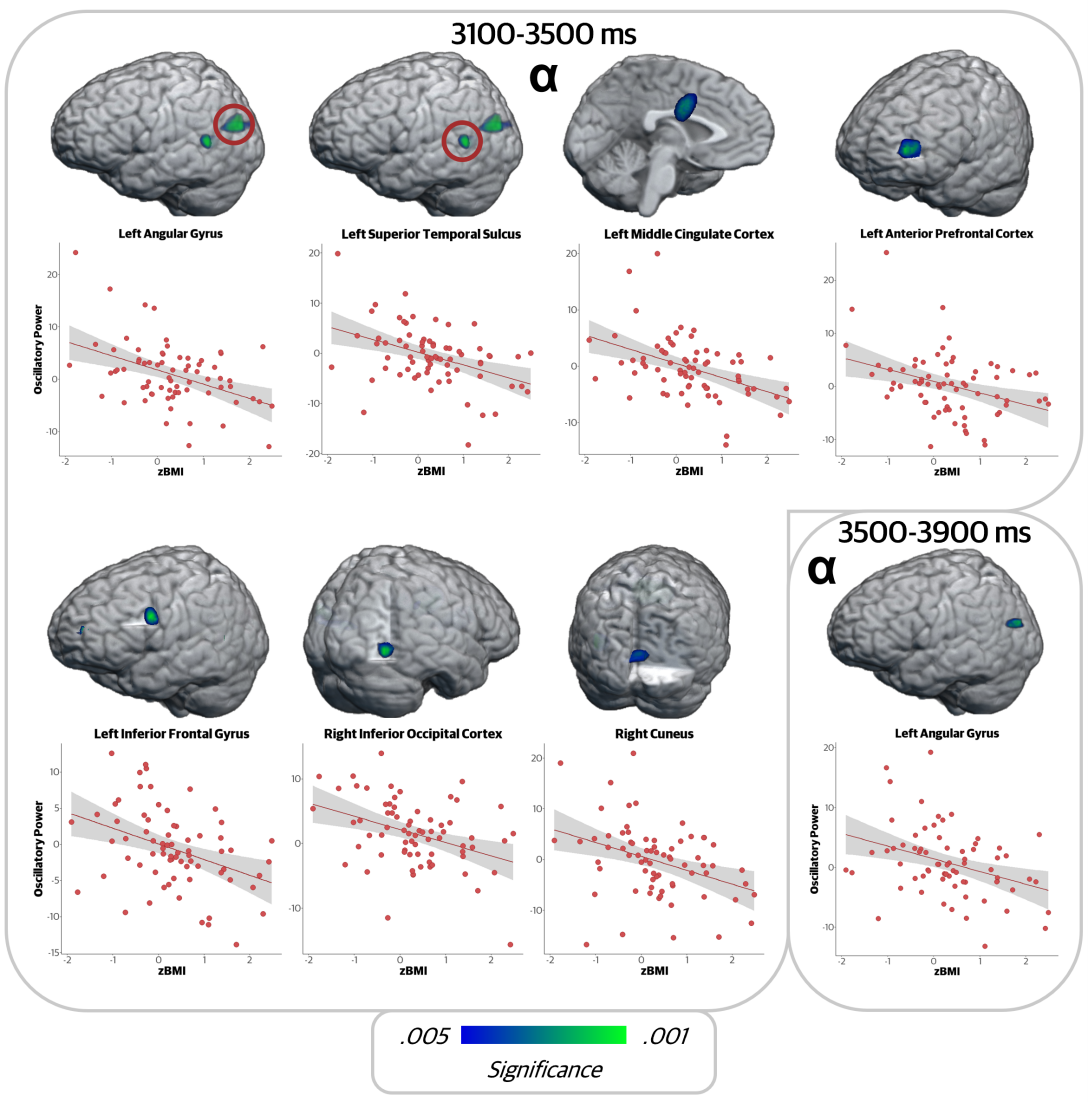
zBMI was associated with alpha oscillatory activity during maintenance. Whole-brain correlation maps per time window and corresponding scatterplots showing relationship at the peak voxel between alpha oscillations during maintenance and zBMI. The x-axis displays zBMI values and the y-axis represents oscillatory power in units of pseudo-*t*. In all regions, there was a negative relationship between zBMI and oscillatory alpha power.

In the two alpha maintenance windows (3100 to 3900 ms), there was again a negative correlation between zBMI and alpha oscillatory power in several regions (Fig. 5). In the first window, significant correlations were observed in the left angular gyrus (*r* = -.434, *p* < .001), left posterior superior temporal sulcus (*r* = -.418, *p* < .001), left middle cingulate cortex (*r* = -.449, *p* < .001), left anterior prefrontal cortex (*r* = -.362, *p* = .002), left inferior frontal gyrus (*r* = -.382, *p* = .001), right inferior occipital cortex (*r* = -.381, *p* = .001), and right cuneus (*r* = -.369, *p* = .002). In all of these regions, alpha oscillations during maintenance became stronger (i.e., more negative) with increasing zBMI values. Finally, in the second maintenance window (3500 to 3900 ms), the relationship in the left angular gyrus persisted (*r* = -.355, *p* = .002).

### 3.4 Exploratory analyses examining neurobehavioral relationships

To examine the functional relevance of the regions identified in the whole-brain models, we conducted an exploratory analysis to determine whether the oscillatory activity that was related to zBMI was also related to performance on the working memory task. Briefly, we performed partial correlations controlling for zBMI between behavioral metrics (i.e., accuracy, reaction time) and oscillatory activity (i.e., pseudo*-t*) at the peak voxel of each cluster identified in the whole-brain correlational analyses. Note that due to the exploratory nature of this analysis, we did not account for multiple comparisons.

**Fig. 6.**
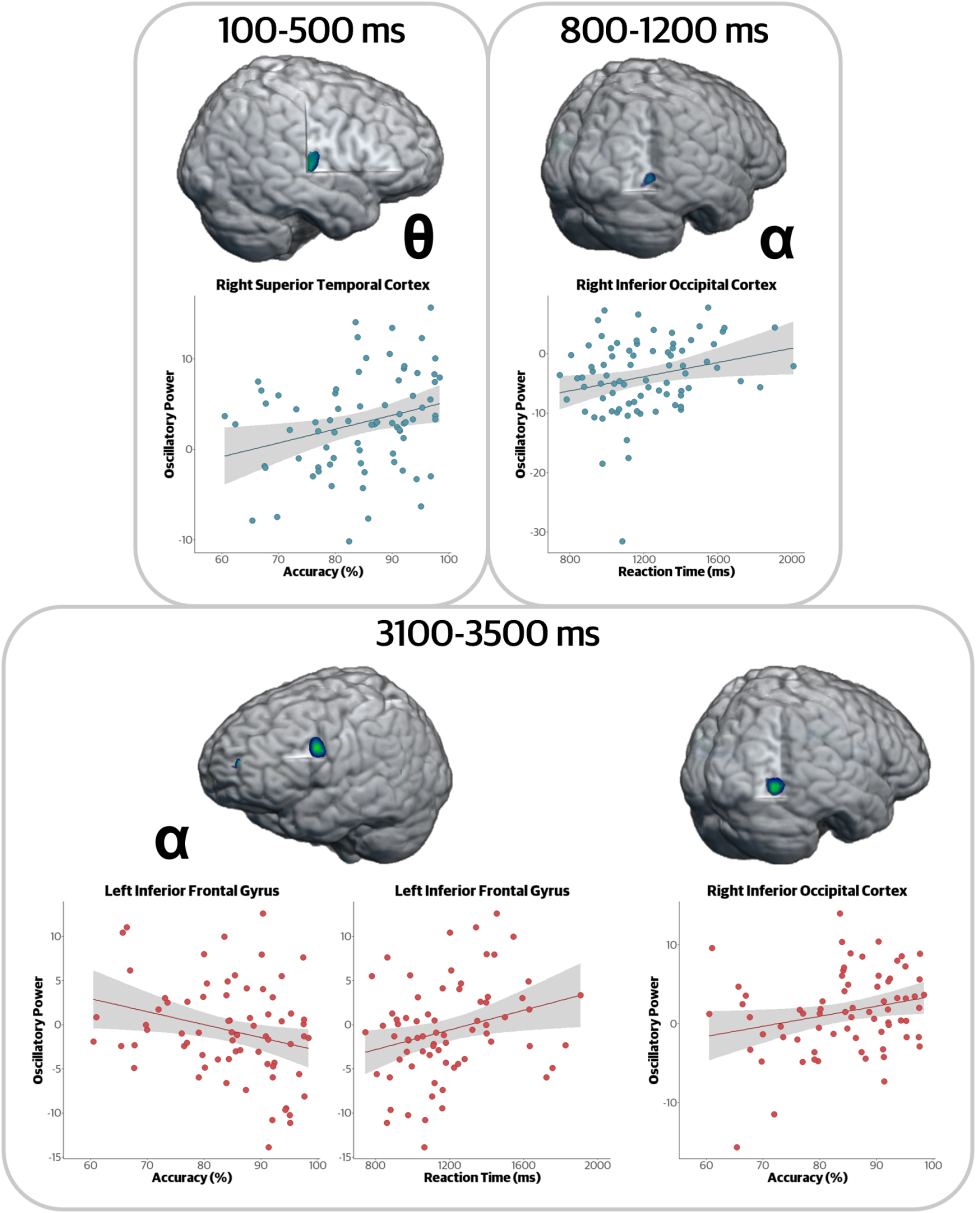
Exploratory brain–behavior correlations. Partial correlations controlling for zBMI between task behavior (accuracy and reaction time) and oscillatory activity were significant at four different clusters identified in the whole-brain analysis. Clusters from the encoding period are displayed in the top panels, with maintenance on the bottom. (Top left) Stronger theta in the right temporal cortex was associated with higher accuracy. (Top right) Stronger alpha (i.e., more negative relative to baseline) in the inferior occipital was associated with faster reaction time. (Bottom left) Stronger alpha (i.e., more negative) in the left inferior frontal gyrus was associated with better accuracy and faster reaction time. (Bottom right) Stronger right occipital alpha was associated with worse accuracy. Y-axes show oscillatory power (pseudo-*t* units), and x-axes show either accuracy or reaction time.

Our findings indicated that the strength of neural oscillations was associated with working memory performance in four regions (Fig. 6). First, in the encoding period, stronger theta responses (i.e., more positive) were associated with higher accuracy (*r* = .309, *p* = .007), and stronger alpha responses (i.e., more negative) in the right inferior occipital cortex were related to faster reaction times (*r* = .250, *p* = .027). During maintenance, stronger left inferior frontal alpha responses predicted better accuracy (*r* = -.287, *p* = .016) and faster reaction times (*r* = .277, *p* = .020), while in the right inferior occipital cortex, stronger alpha predicted lower accuracy (*r* = .272, *p* = .023).

## 4 Discussion

In this study, we used a modified Sternberg working memory paradigm and high-density MEG to study the relationship between age- and-sex-corrected measures of body mass and the neural dynamics supporting subprocesses of verbal working memory (i.e., encoding versus maintenance). Accurate and efficient encoding and maintenance abilities are essential to working memory performance, and it is known that typically developing youth recruit neural resources in different ways compared with adults (Embury et al., 2019; Jolles et al., 2011; Thomason et al., 2009). However, the extent to which the neural oscillatory activity underlying these processes is altered in youth with obesity is drastically understudied. Our key findings add to this literature, suggesting that even subclinical indices of obesity are moderately associated with process-specific alterations to the oscillatory dynamics in both theta and alpha frequency ranges. These findings and their implications are discussed below.

Our first major finding was that zBMI was negatively associated with theta oscillatory activity in brain regions involved in language and memory during the encoding phase. These regions included posterior areas of the superior temporal gyri, which have also been implicated in verbal working memory processes, particularly in the left hemisphere, with multiple studies suggesting these regions may support a sort of phonological loop that would be active during encoding (Diedrich et al., 2025; Embury et al., 2019; Heinrichs-Graham & Wilson, 2015; Killanin et al., 2024a, 2024b, 2025; Proskovec et al., 2018; Rottschy et al., 2012), due in part to subvocal repetition of letters as an encoding strategy. In the present study, our findings expand on this literature by suggesting that participants with higher zBMIs may exhibit weaker theta oscillations (i.e., a smaller increase in power from baseline) during early encoding in bilateral superior temporal regions. We also observed a similar relationship between theta activity and zBMI in the left precentral gyrus. The conversion of visual information into phonological representations, called phonological recoding, is an important step in loading visually presented information into verbal working memory stores (Palmer, 2000), and studies have suggested primary motor cortex activity as a major neural correlate for phonological recoding. For example, patients with cortical damage resulting in impaired speech production were able to perform phonological judgments on auditorily but not visually presented stimuli (Papagno et al., 2008; Vallar, 2006), and transcranial magnetic stimulation of the primary motor cortex of healthy adults elicited larger motor evoked potentials during phonological tasks on visually presented stimuli than auditory stimuli (Romero Lauro et al., 2020). Further, areas near the precentral gyrus, such as the premotor cortex, are also active during verbal working memory as part of the articulatory loop and control of the temporal maintenance of serial order during subvocal rehearsal (Henson et al., 2000). Though our task did not require participants to maintain a temporal order of the stimuli, subvocal rehearsal strategies would inherently necessitate the grouping of letters into some arbitrary order. Our findings indicate that regions important for phonological processing and encoding (Baddeley, 2002; Baddeley & Hitch, 1974; D’Esposito et al., 1995) may also be functionally affected by increases in body mass. Finally, a recent MEG study also found a relationship between blunted theta oscillations and increases in zBMI during a matrix reasoning task (Ward et al., 2024). Deficits in cognitive performance related to altered theta oscillations have also been reported in people with autism (Larrain-Valenzuela et al., 2017), schizophrenia (Schmiedt et al., 2005), youth with high levels of psychosocial distress (Schantell et al., 2024), chronic cannabis use (McDonald et al., 2024, 2025; Rangel-Pacheco et al., 2021; Schantell et al., 2022; Springer et al., 2021), HIV-related cognitive impairment (Arif et al., 2020; Lew et al., 2018; Wiesman et al., 2018; Wilson et al., 2015), and Alzheimer’s disease (Meehan, Embury, et al., 2023; Meehan, Schantell, et al., 2023; Wiesman et al., 2022, 2024; Wiesman, Murman, et al., 2021). Together, this suggests not only that these population-level neural dynamics are critical to cognitive function and mental health, but that they may also be sensitive to perturbation by biological or environmental factors. That said, more work is needed to understand the potential mechanisms by which weight-related disease processes impact cognition, or ways in which cognitive ability may impact the development of obesity.

In contrast to our theta findings, we found that *stronger* (i.e., a greater decrease in power from baseline) alpha oscillations during encoding were associated with higher zBMI, which was observed primarily in occipital regions. Most notably, alpha oscillations in the left superior occipital cortex were significantly correlated with zBMI throughout the encoding period. Several M/EEG studies have suggested that alpha oscillations are a marker of visuospatial attention function, such that greater decreases in power from baseline in occipital regions contribute to heightened attention to a particular stimulus (Gould et al., 2011; Klimesch, 2012; McCusker et al., 2020; Son et al., 2023; Wiesman et al., 2019). Thus, our findings of stronger alpha oscillations (i.e., greater decreases in power relative to baseline) in these regions for those with higher zBMIs may suggest increased deployment of visuospatial attention, perhaps to compensate for the weakened theta responses during early encoding.

In addition, during the maintenance phase, we observed that higher zBMIs were associated with altered left-lateralized alpha activity in prefrontal and posterior temporal areas. A widespread decrease in baseline-relative alpha band activity beginning in the occipital cortices and progressing anterior into temporal and frontal regions during maintenance has been well characterized in working memory tasks in adults and youth, not only in verbal paradigms (Embury et al., 2018, 2019; Heinrichs-Graham et al., 2021; Heinrichs-Graham & Wilson, 2015; Killanin et al., 2022, 2024a), but also in spatial (Proskovec et al., 2018) and numerical paradigms (Koshy, Wiesman, Proskovec, et al., 2020). Our findings corroborate this literature and extend it, as we found increased alpha oscillatory activity (i.e., more negative relative to baseline) in youth with higher zBMI. In a sample of healthy aging adults, Springer et al. (2023) used a similar verbal working memory paradigm and found that alpha/beta activity increased as a function of age in left prefrontal regions. The authors found that age was not associated with accuracy, and the authors hypothesized that this reflected a compensatory mechanism by which increased neural recruitment was utilized to offset potential age-related declines in cognitive performance (i.e., Compensation-Related Utilization of Neural Circuits Hypothesis (CRUNCH; Reuter-Lorenz & Cappell, 2008; Springer et al., 2023). A similar mechanism could be at work in those with higher zBMI, whereby increased recruitment of alpha activity during encoding and maintenance may be coordinated to overcome behavioral deficits in working memory performance. The lack of association between task performance and zBMI may indicate that the compensatory increase in alpha activity is adequate, but in a sample of youth with severe obesity, these mechanisms may break down and could result in poorer performance as the clinical severity increases.

The results of our exploratory analyses lend partial support to the interpretation that the stronger alpha responses (i.e., more negative) are generally a mechanism to compensate for blunted theta oscillations in youth with higher zBMI. In the encoding stage, individuals with the weakest theta responses tended to have the highest zBMIs, and weaker theta predicted poorer working memory performance. However, stronger alpha responses tended to predict better performance, and those who had the strongest alpha responses tended to have higher zBMIs. However, it should be noted that the right inferior occipital cortex appears to be an exception among these clusters, in that stronger alpha responses (i.e., more negative values) were associated with higher zBMI and lower accuracy. As the initial decrease in baseline-relative alpha power progresses anteriorly, increases in power relative to baseline emerge in occipital areas, which is thought to inhibit the processing of irrelevant or distracting stimuli (Bonnefond & Jensen, 2012; Heinrichs-Graham & Wilson, 2015). Thus, it is possible that youth with elevated body mass may be less effective in engaging such inhibitory processing in the occipital cortex, which may impact their ability to adequately maintain relevant information in temporary stores.

Finally, we found that age was strongly associated with behavioral performance on the task. This is consistent with previous developmental investigations from our group, in which older participants were faster to respond and more accurate on verbal working memory tasks (Embury et al., 2019; Killanin et al., 2022, 2024a, 2024b). Adolescence is characterized as a critical period for the development of higher-order cognition, wherein constituent processes of executive function, such as working memory, undergo substantial experience-dependent refinement (Knudsen, 2004; Larsen & Luna, 2018), largely due to the maturation of neural circuitry through processes such as synaptic pruning and axonal myelination (Spear, 2000). Thus, along with this refinement of working memory ability, older youth may learn to employ more efficient strategies for loading and retaining information in memory. It is important to note, however, that results from a recent large-scale study that integrated multiple measures in an investigation of the development of executive function suggest that age-related improvements in executive function are domain general (Tervo-Clemmens et al., 2023). However, given our neural findings, we were somewhat surprised by the lack of a relationship between zBMI and behavioral performance, but this could simply reflect effective compensatory activity as noted above.

Before closing, it is important to note the limitations of this study and discuss potential directions for future work. First, the parent study (DevMIND) did not specifically recruit youth with obesity, and thus the present study was not adequately powered to stratify the participants into groups (i.e., those with and without obesity). As such, future work would benefit greatly from recruiting youth with a clinical diagnosis and directly contrasting them with a healthy-weight group. Second, we focused on verbal working memory processing, and future studies may consider different tasks to determine the specificity with which body mass is related to neural oscillatory activity; that is, studies should probe whether differences in activity associated with obesity are affecting an underlying cognitive component to working memory such as visual attention, or whether these effects are indeed specific to verbal working memory; some recent work suggests they may be more domain general (Li et al., 2023, 2024; Ward et al., 2024).

Despite these limitations, our findings suggest that deviations from normal body mass index in youth are associated with aberrant neural oscillatory activity serving working memory subprocesses. As previously discussed, childhood obesity is a serious global issue that is not only persisting but is also growing in severity. The long-term consequences of early life obesity to individual health are only beginning to be examined, with impacts to cognition and cognitive development in particular being poorly understood. Thus, it is critical that future investigations employ rigorous methodologies to study obesity’s impacts on the developing brain. The present study takes an important step toward understanding the possible mechanisms linking body mass and brain function. The consistency of results in this healthy pediatric sample provides compelling evidence to suggest that even a relatively narrow range of deviation from healthy body mass can impact neurophysiology. Determining the extent to which severe obesity alters brain function, and whether this apparent linear trajectory holds in youth with more severe obesity, is an important avenue for future work expanding the pediatric obesity literature. Such work will help illustrate the importance of obesity prevention and treatment, and may inform intervention targets or policy initiatives to help reduce the individual and societal burdens of obesity.

## Supplementary Material

Supplementary Material

## Data Availability

The data used in this article will be publicly available through the COINS framework (Dev-MIND; https://coins.trendscenter.org/).
