# Supplementary material for: zBMI scales with multispectral alterations in the neural oscillatory dynamics serving verbal working memory in youth
Source: Imaging Neurosci (Camb). 2025 Jul 9;3:IMAG.a.78. doi: 10.1162/IMAG.a.78 (PMC12330846; doi:10.1162/IMAG.a.78)
Supplement: Supplementary Material [file IMAG.a.78_supp.pdf]

Supplementary Materials

zBMI scales with multispectral alterations in the neural oscillatory dynamics serving verbal working memory in youth

Thomas W. Ward, Abraham D. Killanin, Danielle L. Rice, Grace C. Ende, Erica L. Steiner, Anna T. Coutant, Christine M. Embury, Vince D. Calhoun, Yu-Ping Wang, Julia M. Stephen, Elizabeth Heinrichs-Graham, and Tony W. Wilson

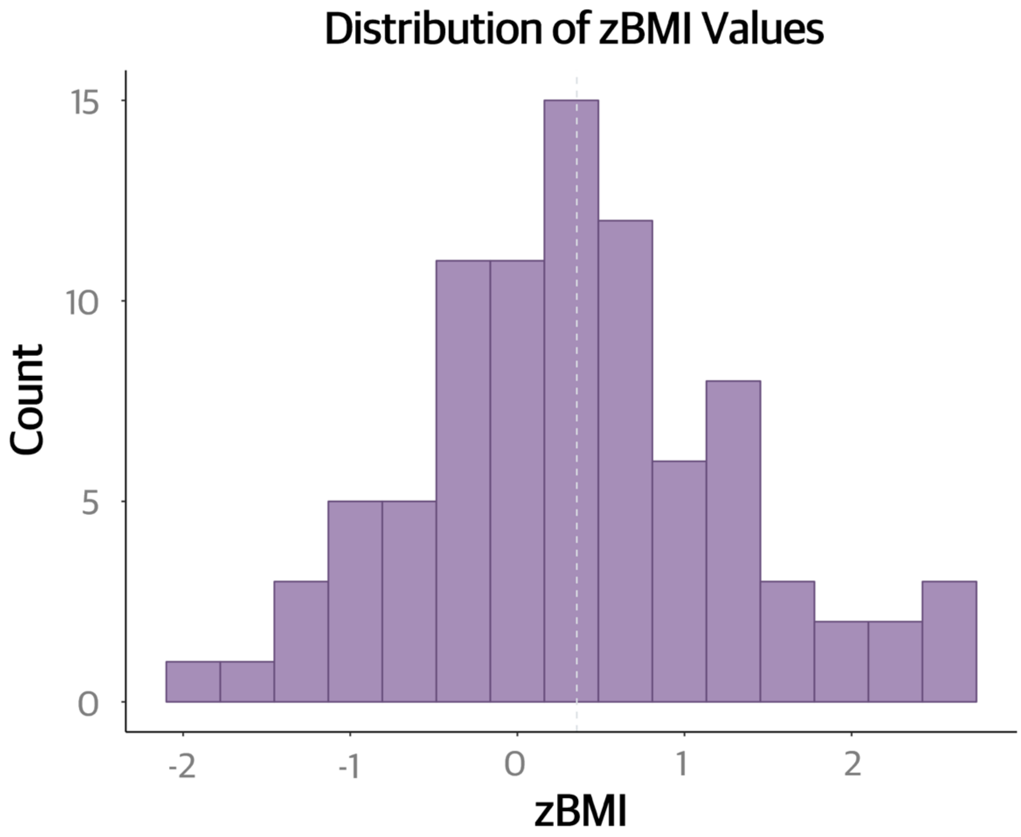

Supplementary Figure 1. Histogram of zBMI distribution across the sample. *Age-and-sex-adjusted BMI (zBMI) is plotted on the x-axis, with count on the y-axis. Dashed grey line depicts the mean zBMI value for the sample (0.36)*
